# The Composition of Saturated Vapor over 1-Butyl-3-methylimidazolium Tetrafluoroborate Ionic Liquid: A Multi-Technique Study of the Vaporization Process

**DOI:** 10.3390/e23111478

**Published:** 2021-11-08

**Authors:** Anatoliy M. Dunaev, Vladimir B. Motalov, Lev S. Kudin

**Affiliations:** Research Institute of Thermodynamics and Kinetics, Ivanovo State University of Chemistry and Technology, 153000 Ivanovo, Russia; v.motalov@gmail.com (V.B.M.); LKudin@yandex.ru (L.S.K.)

**Keywords:** ionic liquids, imidazolium, Knudsen effusion mass spectrometry, vapor composition, ylidene, imidazole

## Abstract

A multi-technique approach based on Knudsen effusion mass spectrometry, gas phase chromatography, mass spectrometry, NMR and IR spectroscopy, thermal analysis, and quantum-chemical calculations was used to study the evaporation of 1-butyl-3-methylimidazolium tetrafluoroborate (BMImBF_4_). The saturated vapor over BMImBF_4_ was shown to have a complex composition which consisted of the neutral ion pairs (NIPs) [BMIm^+^][BF_4_^−^], imidazole-2-ylidene C_8_N_2_H_14_BF_3_, 1-methylimidazole C_4_N_2_H_6_, 1-butene C_4_H_8_, hydrogen fluoride HF, and boron trifluoride BF_3_. The vapor composition strongly depends on the evaporation conditions, shifting from congruent evaporation in the form of NIP under Langmuir conditions (open surface) to primary evaporation in the form of decomposition products under equilibrium conditions (Knudsen cell). Decomposition into imidazole-2-ylidene and HF is preferred. The vapor composition of BMImBF_4_ is temperature-depended as well: the fraction ratio of [BMIm^+^][BF_4_^−^] NIPs to decomposition products decreased by about a factor of three in the temperature range from 450 K to 510 K.

## 1. Introduction

In recent years, ionic liquids (ILs) have become one of the most fast-developing fields of chemistry. This keen interest is due to the unique combination of IL’s properties, which includes the ability to dissolve organic, inorganic and polymer materials together with low vapor pressure at room temperatures [1]. Today, imidazolium-based ILs are the most investigated group of ionic liquids. They are distinguishable from others by quantifiable vapor pressures at 380–500 K and their relatively high decomposition temperatures [2]. This fact gives possibility to the use of these ILs in various heterophase processes such as distillation, chemical gas-phase deposition, etc. Therefore, the investigation of evaporation of the imidazolium-based ILs is particularly important.

It has been experimentally proven that many aprotic ILs evaporate congruently and that their vapors consist of neutral ion pairs (NIPs) [3,4,5,6,7,8]. However, evaporation of a number of aprotic ILs can be accompanied by a partial decomposition of the condensed phase. Some ILs, e.g., those containing chiral center, decompose while heating, and the decomposition processes prevail over evaporation [9]. Decomposition was marked for ILs with high electronegative anions (BF_4_^−^, PF_6_^−^, AsF_6_^−^, SCN^−^, etc.) as well [10,11,12]. The 1-butyl-3-methylimidazolium tetrafluoroborate (BMImBF_4_) is a striking example of such a compound. As opposed to the alkylimidazolium ILs with NTf_2_^−^ anion having a simple vapor composition consisting exclusively of NIPs [6,13] at temperatures below the onset of decomposition, the vapor of BMImBF_4_ contains many other components [14] at quite low temperatures, where any appreciable effects on thermal analysis curves are not evident. However, there is no consensus in the literature about this phenomenon: some works [14,15,16,17,18,19,20,21,22] are concerned with the thermal behavior of BMImBF_4_, and conclude that the no thermal degradation of IL occurred, while at the same time the other authors [23,24,25,26,27] postulate the thermal decomposition of the investigated compound.

**Figure 1:**
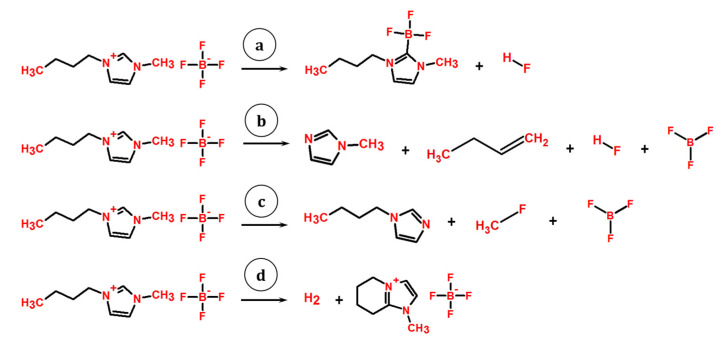
Schemes of BMImBF_4_ decomposition with formation of imidazole-2-ylidene and HF (**a**); 1-methylimidazole, 1-butene, HF, and BF_3_ (**b**); 1-butylimidazole, fluoromethane, and BF_3_ (**c**); H_2_ and bicyclic IL (**d**).

In a recent paper [10], the evaporation mechanism of BMImBF_4_ was investigated using NMR analysis and mass spectrometry. The authors established the competitive vaporization and thermal decomposition of IL ruled by the sample surface area to volume ratio. The main decomposition route proposed in [10] was the formation of imidazol-2-ylidene (Figure 1a) through an Arduengo carbene [28] by cation-anion interaction. Previously, borane-substituted imidazol-2-ylidene was synthesized in vacuo from BMImBF_4_ by Taylor et al. [29]. Some researchers [10,29] gave the NMR and mass spectra of pure ylidene and found that its vapor pressure was sufficiently higher than that of IL at the same temperatures. The evaporation of BMImBF_4_ can also be accompanied by the formation of imidazoles [26,27]. These routes are the formation of 1-methylimidazole, 1-butene, HF, and BF_3_ (Figure 1b), and 1-butylimidazole, fluoromethane, and BF_3_ (Figure 1c). The pyrolysis-gas chromatography experiments [26,27] were carried out at much higher temperatures than that of decomposition (873 K and 823 K in refs. [26] and [27] respectively). The authors [26] concluded that the ratio between these two routes is close to 1:1. According to Ohtani et al. [27] the reaction with 1-methylimidazole formation is preferred. Formation of ethylimidazole from similar IL BMImPF_6_ was observed by KEMS in Ref. [12]. Authors [12] noted the influence of orifice size on vapor composition: the larger the orifice, the larger the contribution of the BMImPF_6_ vapor species. The investigation of ILs with cyano-functionalized anions [11,30], which are close to the object under study, showed one more degradation route by the intrinsic cyclization (Figure 1d) of the butyl group on the C1 atom.

In a recent paper [31] the kinetic model of maximum operation temperature (MOT) was applied to BMImBF_4_ IL. This model defines the temperature at which a mass loss of 1%, which can be attributed to thermal decomposition, occurs as a function of variable application time. It was found that MOT decreased exponentially with increasing application time (466 K at 1 h and 348 K at 1 year). The authors [31] also studied the vapor composition of BMImBF_4_. The decomposition products according to routes **b** and **c** (here and further designations as in Figure 1) were found in vapor. Additionally, the formation of imidazole and 1-butene from 1-butylimidazole was suggested and traces of imidazole were found.

Mass spectroscopy [10,14] revealed the partial decomposition of initial IL according to way **a** and did not find any traces of imidazoles. It should be noted that in a recent paper [12] with the analogous BMImPF_6_ IL, none of the ylidenes were registered, whereas the ethylimidazole was observed in vapor.

The available thermodynamic studies of BMImBF_4_ were carried out without any analysis of the gas phase composition [17,18,19]. As a result, the vapor pressures of BMImBF_4_ obtained in these works disagree by some orders of magnitude. Therefore, the comprehensive analysis of the vapor composition of the mentioned group of ILs is mandatory in thermodynamic investigations.

This work is a multi-technique study of the BMImBF_4_ evaporation carried out with the use of Knudsen effusion mass spectrometry (KEMS), IR and NMR spectroscopy, thermal analysis, gas-phase chromatography–mass spectrometry (GCMS), and quantum chemical modelling. The main goals are to determine the composition of saturated vapor over BMImBF_4_ and to clarify the routes of thermal decomposition while heating.

## 2. Experimental

Thermal analysis of the samples (98% purity, Sigma-Aldrich, St. Louis, MO, USA) was performed on a synchronous thermal analysis instrument Netzsch STA 449 F3 Jupiter (NETZSCH-Gerätebau, Selb, Germany) in the temperature range of 20–500 °C at a speed of 5 °C/min in nitrogen atmosphere. The device has high sensitivity with a resolution of 0.1 μg. In parallel with the data on weight loss, the temperature dependences of the thermal effects were recorded at the resolution of 1 μW.

IR spectroscopic measurements were carried out on a Bruker Tensor 27 (Bruker AXS, Madison, WI, USA) spectrometer with Fourier transform. The operating frequency range was 370–4000 cm^−1^ with a resolution of 1 cm^−1^. The instrument makes it possible to obtain both the spectra of the condensed phases and the temperature dependences of the absorption for the gas phase.

NMR spectra ^1^H, ^13^C, ^11^B, ^15^N in DMSO-*d*_6_ at *T* = 22 °C and *T* = 70 °C were recorded by a Bruker Avance 500 (Bruker AXS, Madison, US) spectrometer with 5 mm TBI 1H/31P/D-BB z-GRD sensor. A working frequency for ^1^H was 500.17 MHz, ^13^C—125.77 MHz, ^11^B—160.47 MHz, ^15^N—50.68 MHz. ^13^C NMR spectra were obtained using broadband proton decoupling (WALTZ 16). ^15^N chemical shift measurements were made based on two-dimensional HMBC ^15^N-^1^H spectra. The solvent signal (DMSO-d6) was used as a reference for ^1^H, ^13^C spectra; BF_3_OEt_2_ was used for ^11^B; nitromethane was used for ^15^N. The signal assignment in spectra of objects under study was performed on a base of literature data [32,33,34,35,36] and NMR-prediction instruments.

A magnetic sector mass spectrometer MI1201 (PO “Electron”, Sumy, Ukraine) coupled with a Knudsen cell was used for vapor analysis. Neutral vapor species were studied using a combined ion source operating in electron-ionization (EI) mode. A detailed description of the apparatus is given elsewhere [37,38,39].

The GCMS experiments were carried out on a Shimadzu GCMS QP2010 Ultra (Shimadzu, Kyoto, Japan). Each sample was analyzed in programmable mode: a column temperature was kept at 100 °C during 5 min, after that the sample was heated with a speed of 5 °C/min up to 250 °C. Two types of columns were used: polar (Agilent DB-17MS capillary column) and non-polar (Zebron ZB-5MS capillary column).

## 3. Computational Details

The molecular structure of conformers of the neutral ionic pair [C_4_mim^+^][BF_4_^−^] and the cation [C_4_mim^+^] has been studied by the density functional theory method (pure B3LYP, B3LYP with D3 version of Grimme’s dispersion [40], long-range-corrected version of B3LYP using the Coulomb-attenuating CAM-B3LYP functional [41], as well as hybrid functional of Truhlar and Zhao M06 [42]) with the use of the Dunning’s correlation consistent triple basis sets cc-pVTZ [43]. All calculations were carried out using the Gaussian 09 package [44]. Comparison of IR spectra of computed molecules with experiment revealed a more accurate description of molecular structure by the CAM-B3LYP functional, which is used in all calculations.

**Figure 2:**
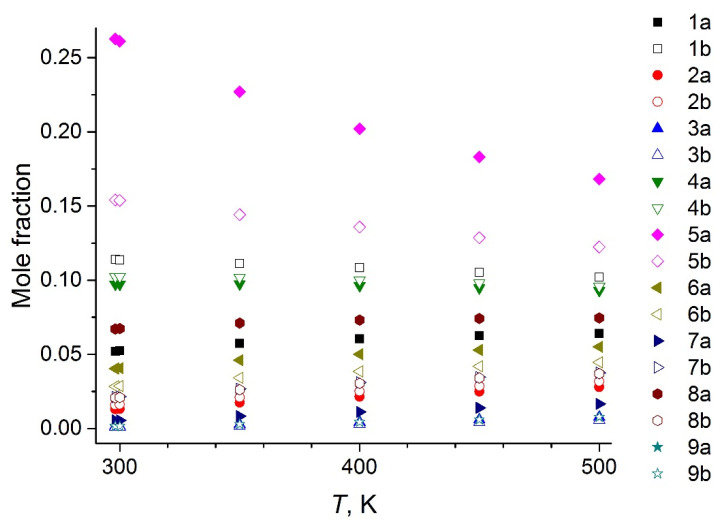
Computed vapor composition of the BMImBF_4_ conformers at 300–500 K (the structures corresponding to designations see in Supplementary Materials, Figure S1).

**Figure 3:**
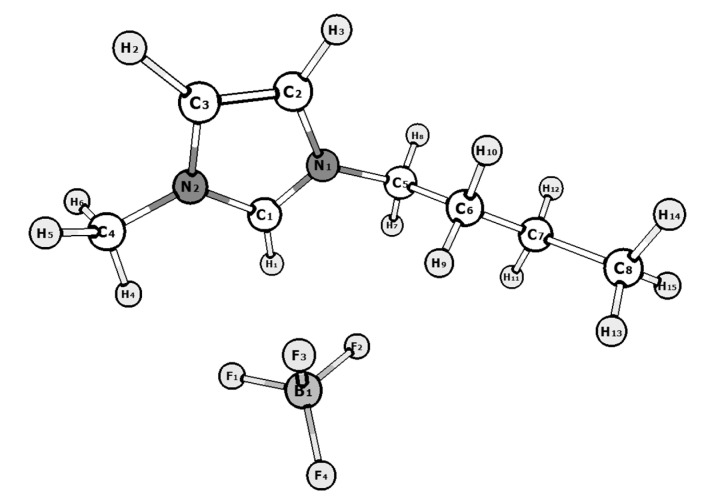
Structure of conformer **5a**.

The nine most energetically preferable conformers of the [BMIm^+^] cation were selected on a basis of conformational analysis. The structures of the BMImBF_4_ conformers are depicted in Figure S1 (see Supplementary Materials). Each cation conformer can exist together with the [BF_4_^−^] anion in two forms: “close” (denoted as “a”) and “open” (denoted as “b”). The equilibrium mole fractions of the conformers in the temperature range of 300–500 K found on a basis of their relative free energies calculated by DFT are presented in Figure 2. The conformer **5a** due to its lowest energy dominates in vapor at experimental conditions (400–500 K). Its structure is shown in Figure 3.

The IR spectra were modeled in the following manner: the intensities of vibrations of the conformers were multiplied by the corresponding mole fractions and are summarized through observed IR range (40–400 cm^−1^).

## 4. Results

### 4.1. Thermal Analysis

**Figure 4:**
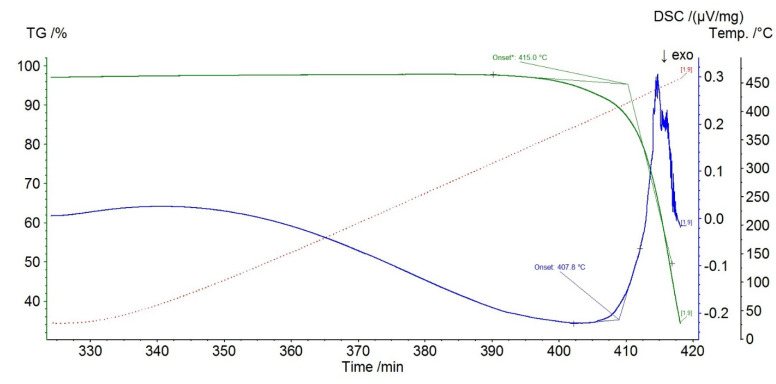
DTA results for BMImBF_4_.

The decomposition temperature *T*_dec_ = 685 K was found as the average between the mass loss and DSC data (Figure 4). This value is quite close to that of 679 [31], 697 K [45], 712 K [46], 633–723 K [47] whereas the other literature data are somewhat lower: ~640 K [26], 653 K [48], and 634 K [23]. None of additional effects before decomposition were found.

### 4.2. NMR Analysis

**Figure 5:**
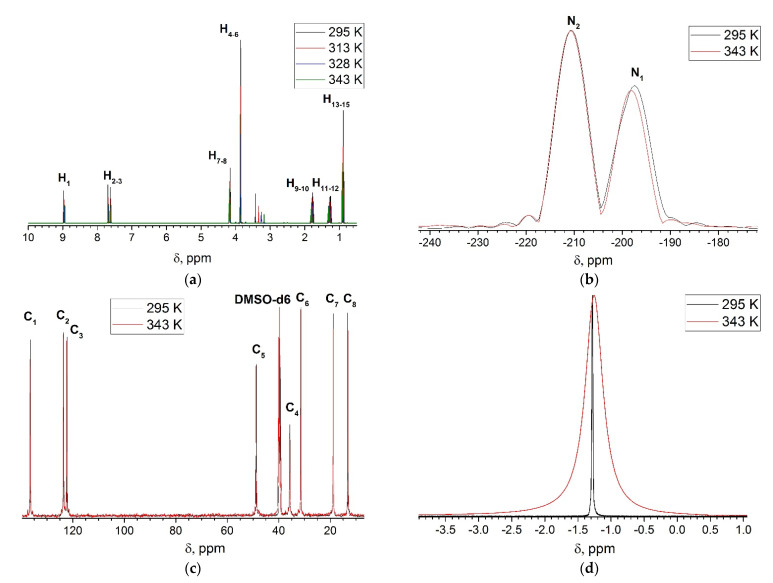
NMR spectra of BMImBF_4_ at 295–343 K: (**a**) ^1^H; (**b**) ^15^N; (**c**) ^13^C; (**d**) ^11^B. Atom numbering is in accordance with Figure 3.

None of structural changes on heating were revealed (Figure 5). The only effect of temperature change was marked for ^11^B. It is a broadening of B1 peak due to increasing of anion motion around cation upon heating. The obtained ^1^H spectra are in agreement with those from [32,34,35,36] and disagree with data from [33]. A possible source of such discrepancy is that, in the latter case, the authors used non-commercial self-synthesized samples and therefore some impurities may not be removed. The theoretical ^1^H NMR spectra of [BMIm^+^][BF_4_^−^] NIP, imidazol-2-ylidene, and bicyclic IL methyl-4C-imidazolium tetrafluoroborate were obtained by quantum chemical calculations at CAM-B3LYP/aug-cc-pVTZ level of theory. The chemical shift scale was calibrated by tetramethylsilane (TMS). The comparison of theoretical (Figure S2, Supplementary Materials) and experimental spectra corresponds to the structure of [BMIm^+^][BF_4_^−^] NIP. The comparison of the obtained ^1^H and ^13^C spectra with those of imidazole-2-ylidene [10], 1-butylimidazole [49], and 1-methylimidazole [50] indicates the absence of traces of these decomposition products in initial IL.

### 4.3. KEMS

**Figure 6:**
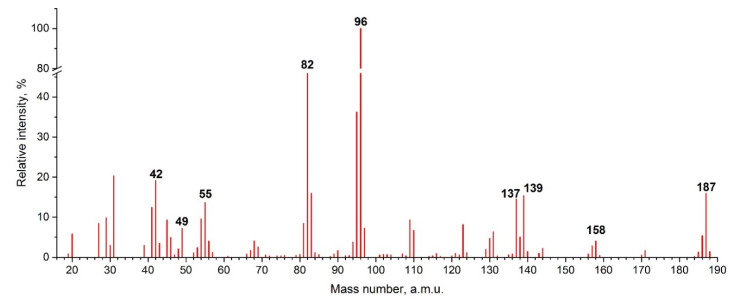
EI background subtracted mass spectrum of BMImBF_4_ at 472 K.

Mass spectrometric experiments were performed in the temperature range of 424–514 K, much below the decomposition temperature (685 K) found by the thermal analysis. The background subtracted mass spectrum recorded at 472 K and the energy of ionizing electrons of 40 eV is shown in Figure 6. In contrast with alkylimidazoilum ILs with NTf_2_^−^ anion [6,13] the obtained mass spectrum has some prominent features. First, the parent cation with *m*/*z* = 139 has very low relative intensity; second, the lightweight fragment ions have high intensities with dominating ion with *m*/*z* = 96; third, the ions with the higher mass than that of the parent cation were also registered (*m*/*z* = 158, 187).

**Figure 7:**
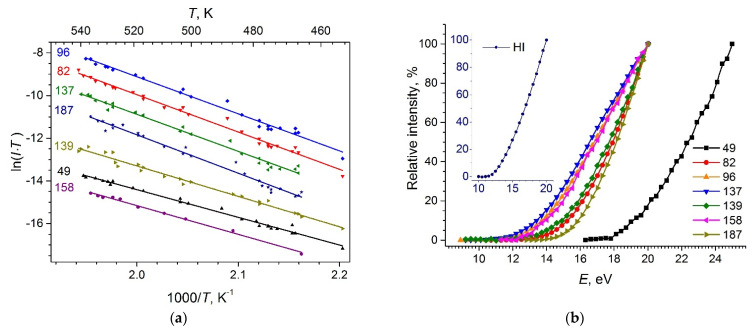
Temperature dependencies of ion currents (**a**) and ionization efficiency curves (**b**). The intensities of ions with *m*/*z* = 139 and 187 are scaled by a factor 0.25; *m*/*z* = 49 by 0.14, and *m*/*z* = 158 by 0.11 to be more illustrative.

The temperature dependencies of ion currents in the form ln(*IT*) vs. 1/*T* and the ionization efficiency curves were measured for the most intense ions (Figure 7). The ion appearance energies (*AE*) obtained from the ionization efficiency curves by a linear extrapolation method together with the slopes of the temperature dependencies are listed in Table 1. The energy scale was calibrated using the background signal of HI^+^ (*IE*(HI) = 10.38 eV [51]).

**Figure 8:**
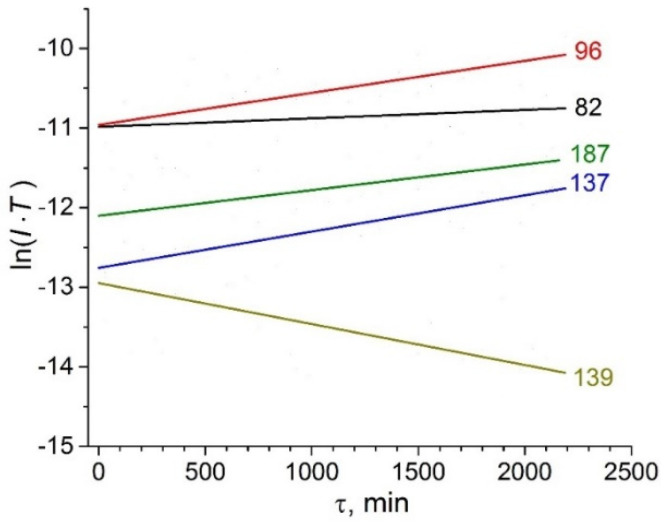
Time dependencies of ion currents at 480 K.

The temporal dependencies of the ion currents for ions with *m*/*z* = 82, 96, 137, 139, and 187 were measured during 36 h at *T* = 480 K and are shown in Figure 8. The ion currents for *m*/*z* = 96, 137, and 187 increase in time, the ion current for *m*/*z* = 82 is practically time-independent, while the ion current for *m/z* = 139 decreases in time.

The mass spectrum obtained in this work considerably differs from that in [14]. In the latter work the major peak in mass spectrum was *m*/*z* = 139, with co-dominating *m*/*z* = 82. Despite the ion with *m*/*z* = 158 was registered, none of the heavier ions were found. It should be mentioned that the evaporation in [14] was carried out from the open surface in Langmuir conditions while in our work the evaporation was performed in Knudsen conditions.

**Figure 9:**
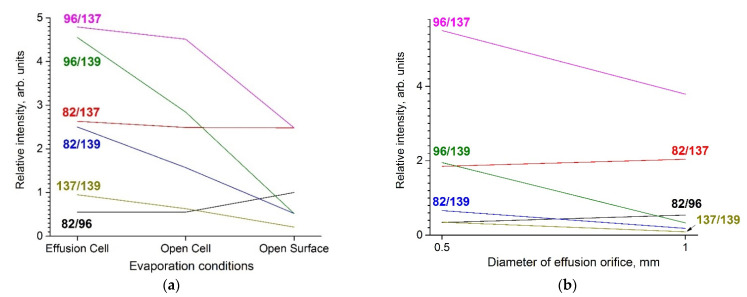
Intensity ratio of ion currents of BMImBF_4_ (**a**) and BMImPF_6_ [12] (**b**).

To clarify the influence of evaporation conditions on vapor composition, the additional experiments on BMImBF_4_ evaporation from the open Knudsen cell (intermediate between Knudsen and Langmuir conditions) and from the entirely open surface of IL (Langmuir conditions) were carried out. The recorded mass spectra for the selected peaks are given in Table 2. The mass spectrum from the open surface is very close to that obtained in [14]. The tendency of increasing of the intensity of the parent cation (*m*/*z* = 139) is observed in the effusion cell—open cell–open surface series, while the intensity of ions with *m*/*z* = 82, 96, 137 decreases in the same series. The ion with *m*/*z* = 187 was found only in Knudsen conditions. In work [12] the same effect of the mass spectrum dependence from the area of the effusion orifice was reported for the BMImPF_6_. The changes in the mass spectra at different evaporation conditions for BMImBF_4_ and BMImPF_6_ are shown in Figure 9. One can see that the behavior of relative intensities is the same for both ILs and strongly depends on a ratio of effusion area to evaporation area.

### 4.4. IR-Spectroscopy

**Figure 10:**
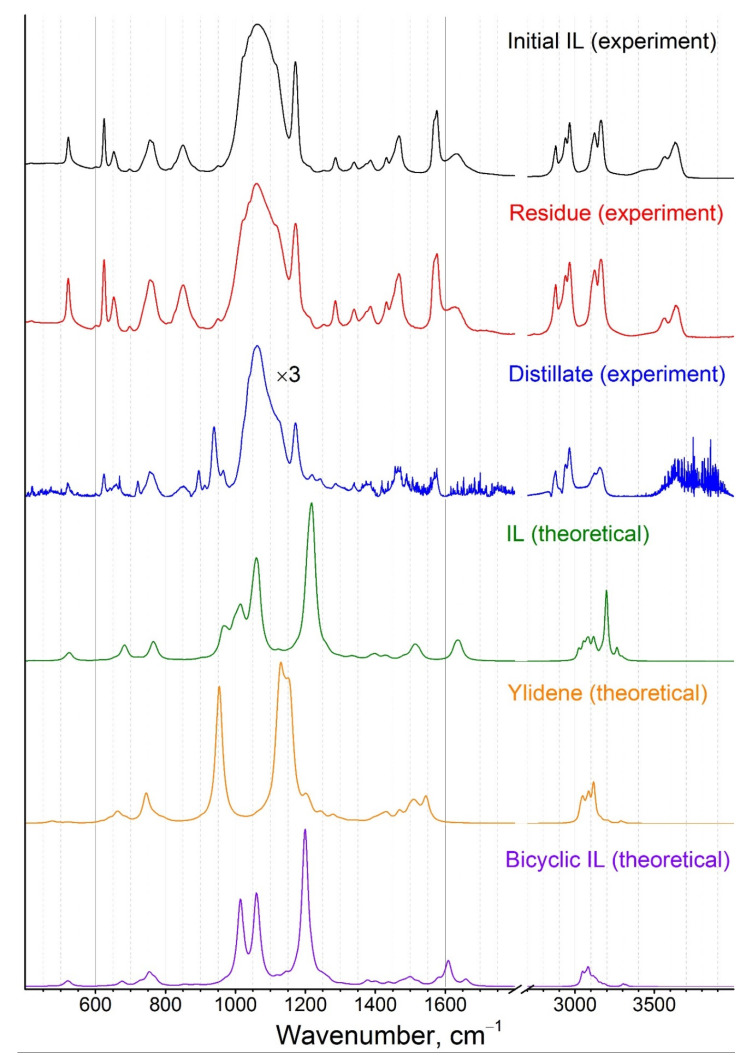
Experimental IR-spectra of condensed phase of BMImBF_4_ and theoretical IR-spectra of BMImBF_4_, imidazole-2-ylidene, and bicyclic IL.

**Figure 11:**
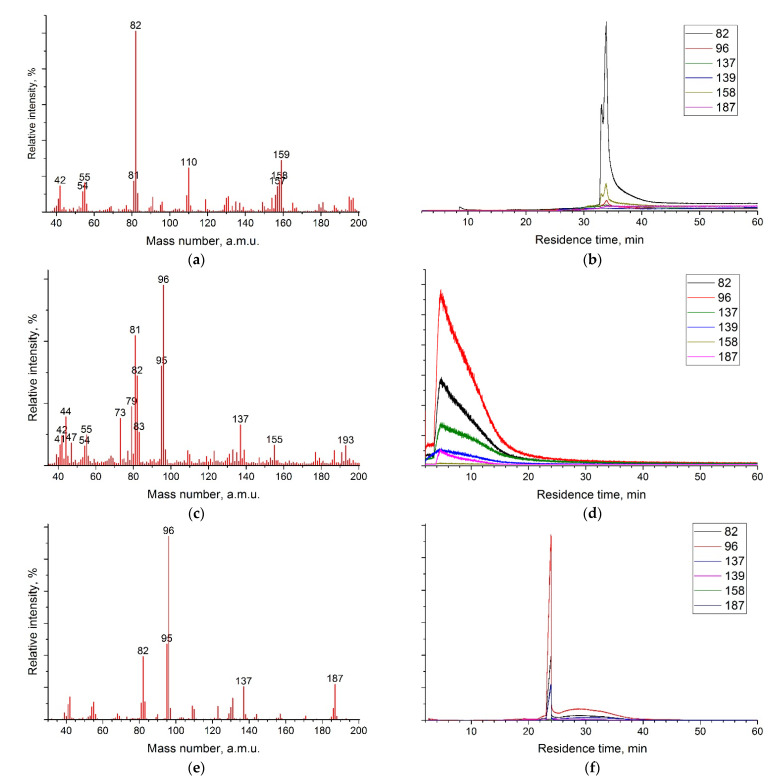
GCMS mass spectra and ion profiles on chromatograms of BMImBF_4_ with polar (**a**,**b**) and nonpolar chromatograph column without (**c**,**d**) and with (**e**,**f**) addition of ethanol.

**Figure 12:**
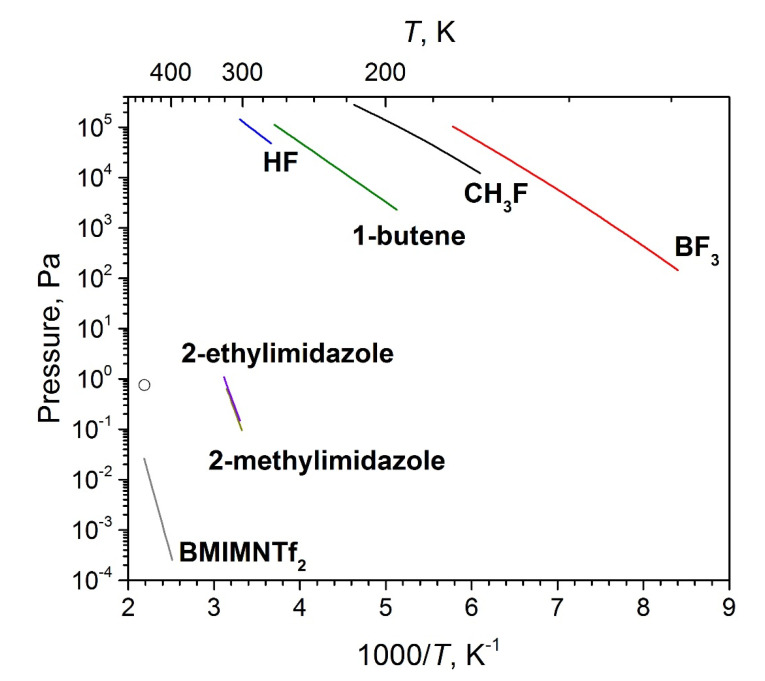
Temperature dependencies of vapor pressures of some decomposition products of BMImBF_4_. Temperature dependence of BMImNTf_2_ is shown for comparison. Assessed pressure of ylidene was marked as a circle.

The IR-spectra (Figure 10) were recorded for the initial IL, the residue after the mass spectrometric experiment, and the distillate collected from the surface of the collimator located in front of the effusion orifice. The analysis of the spectra revealed the absence of any substantial changes in the condensed phase in the Knudsen cell during mass spectrometric experiments, when the sample was heated up to 514 K. However, the IR-spectrum of the distillate had some distinctive features in 800–1000 cm^−1^ region. All obtained spectra were identical to those in [17]. To attribute registered peaks in the spectra a quantum chemical modelling of the vibrational spectrum (CAM-B3LYP/cc-pVTZ level of theory) was performed. All theoretical spectra were calculated at 500 K as a combination of those for conformers (18 conformers in the case of BMImBF_4_; 9 for ylidene; two for bicyclic IL) taking into account their mole fractions (Figure 11 and Figure 12).

Comparison of the theoretical and experimental spectra showed that the initial IL as well as the residue after the KEMS experiment consisted of BMImBF_4_ whereas the distillate contains imidazole-2-ylidene along with BMImBF_4_.

### 4.5. GCMS

The GCMS mass spectra and the ion profiles in BMImBF_4_ chromatograms with a polar and nonpolar chromatograph column are shown in Figure 11. The chromatogram profile on the nonpolar column recorded for the pure undiluted IL was rather broadened (Figure 11d). The most likely explanation is an interaction of BMImBF_4_ with the column material. The addition of ethanol into the sample results in a peak narrowing (Figure 11f). The only peak on the chromatogram pointed out the presence of a single nonpolar compound in vapor.

The chromatogram recorded on the polar column (Figure 11a) had the only peak as well (despite of a slight splitting both peaks have the same mass spectra). Its mass spectrum is characterized by the dominating ion with *m*/*z* = 82 and the sufficiently lower intensity peaks with *m*/*z* = 159, 110, 55, and 42.

## 5. Discussion

To determine the vapor composition of BMImBF_4_ a proper mass spectrum interpretation should be carried out. Molecular precursors of the ions were defined based on the data of the KEMS and GCMS experiments.

The identification of the molecular precursors of the ions was performed on the basis of two principles: (1) the ions from the same molecule usually show close slopes of the temperature dependencies of ion currents, and (2) *AE* of fragment ions increases with decreasing their masses. In addition, for experiments on the Knudsen/Langmuir evaporation, the following statement is true: the ratio of ion currents from the same molecule does not depend on the evaporation conditions.

The parent cation with *m*/*z* = 139 has *AE* (12.4 ± 0.5 eV), which is consistent with the value from [14] (12.8 ± 0.4 eV). This *AE* value is considerably higher than those obtained for the parent cations of prototypical ILs BMImNTf_2_ (9.3 ± 0.3 eV) [12] and EMImNTf_2_ (8.9 ± 0.2 eV) [52] and at the same time it is closer to the *AE* obtained for the similar BMImPF_6_ IL (11.3 ± 0.5 eV) [12]. This fact points out another nature of electron ionization of such class of ILs caused by the stronger cation-anion interaction. As a result, the ion with *m*/*z* = 158 corresponding to BMImF^+^ has appeared. The scheme of this ion formation suggested in [14] includes the intramolecular rearrangement during ionization. *AE* (BMImF^+^/BMImBF_4_) = 11.7 ± 0.5 eV is lower than that of the parent cation. The same situation was observed for origination of BMImF^+^ from BMImPF_6_ (*AE* (BMImF^+^/BMImPF_6_) = 11.1 ± 0.3 eV) [12]. The slopes of temperature dependency of ion current for the ions with *m*/*z* = 158 and *m*/*z* = 139 are very close (Table 1) indicating the origination of the ion with *m*/*z* = 158 directly from NIP. This fact is additionally confirmed by GCMS data on a polar column where the signal with *m*/*z* = 158 was detected. An indirect proof of the origin of the ion with *m*/*z* = 158 from NIP is the constant ratio 158/139 observed with the different sizes of effusion orifice in the experiment with BMImPF_6_ [12].

The ion with *m*/*z* = 49 is BF_2_^+^ having *AE =* 16.9 ± 0.5 eV close to *AE* (BF_2_^+^/BF_3_) = 16 ± 1 eV [53] indicating BF_3_ as a possible molecular precursor of this ion. At the same time, the slope of the temperature dependence for an ion with *m*/*z* = 49 is similar to that for ions with *m*/*z* = 139 and 158 (Table 1). This indicates the second source of origin of the BF_2_^+^ ion from [BMIm^+^][BF_4_^−^] NIP.

The ions with *m*/*z* = 82, 96, 137, and 187 have similar slopes of the temperature dependencies of ion currents. Their *AE* values increases in the *m*/*z* series 137-96-82 corresponding to the abovementioned assignment rule. An exception from this rule is the ion with *m*/*z* = 187 having the highest appearance energy. However, this circumstance can be explained assuming the different ionization process for this ion. Let us demonstrate it basing on the *AE* values of BF_3_. The bond between boron and fluorine is quite strong, even to detach one fluorine atom from boron trifluoride it needs the high energy *AE* (BF_2_^+^/BF_3_) = 16 ± 1 eV [53]. Therefore, the relatively high *AE* value of the ion with *m*/*z* = 187 can be explained by the nature of B-F interaction. A similar situation is observed [54] upon ionization of the B_2_F_4_ molecule with *AE* (BF^+^/B_2_F_4_) < *AE* (BF_2_^+^/B_2_F_4_) < *AE* (B_2_F_3_^+^/B_2_F_4_) due to the different processes for the BF^+^, BF_2_^+^, and B_2_F_3_^+^ ions formation including different co-products. The data of KEMS, GCMS and Knudsen/Langmuir evaporation experiments confirmed a single source of the ions with *m*/*z* = 96, 137, and 187. Comparison of the mass spectra obtained for IL by GCMS and KEMS, and by DIMS [10] for imidazole-2-ylidene showed their qualitative similarity (Table 3). Small quantitative differences can be explained by some additional contribution into these signals from NIP in our work and a possible difference in the ion source constructions as well as different types of mass analyzers. Therefore, one can conclude that imidazole-2-ylidene is the main molecular precursor of these ions. Ylidene has no ionic bond and its ionization mechanism is close to those of inorganic polyhalides (see [55]) where the intensity of the molecular ion is less (or absent at all) than that of the first dissociative ion. That is why there is no molecular ion with *m*/*z* = 206, but the ion with *m*/*z* = 187 is present in the mass spectrum.

The assignment of the ion with *m*/*z* = 82 is rather complicated. Analysis of the temperature dependencies of ion currents and the ionization efficiency curves allows us to assign it to the same neutral precursor as for the ions with *m*/*z* = 96, 137, and 187, i.e., to imidazole-2-ylidene. However, according to GCMS data, the ion with *m*/*z* = 82 is present in the mass spectrum on both the polar and nonpolar columns. The previous studies [3,4] of ILs with the NTf_2_^−^ anion show that the ion with *m*/*z* = 82 is common for alkylimidazolium ILs. This ion can also be formed from 1H-imidazoles via routes **b** and **c** (Figure 1). Analysis of possible evaporation routes according to the schemes depicted in Figure 1b,c was performed on the basis of data from the NIST Mass Spectrometry Data Center [56]. A list of the main peaks in mass spectra for various decomposition processes [56] is given in Table 4. According to route **c** (Figure 1) the intensity of the ion with *m*/*z* = 34 corresponding to CH_3_F should be very strong; the same is expected for the ion with *m*/*z* = 97—the fragment from 1-butylimidazole. However, the KEMS data don’t support this assumption. All main peaks corresponding to route **b** were found in the mass spectrum. The presence of ions with *m*/*z* = 41 and *m*/*z* = 42 in our mass spectrum and their absence in mass spectrum of pure ylidene [10] pointed out the possibility of evaporation of IL by way **b**. Hence the ion with *m*/*z* = 82 has at least three sources: ylidene, 1-methylimidazole, and BMImBF_4_. The most significant results supporting this conclusion were obtained in an isothermal evaporation experiment (Figure 8). The intensity of the ion with *m*/*z* = 82 was almost time-independent, while the intensities of ions with *m*/*z* = 96, 137, 187 increased in time considerably. However, the growth in the intensity of these ions was accompanied by a rapid decreasing of the parent cation signal (*m*/*z* = 139). Therefore, one can assume that at the initial stage the ion with *m*/*z* = 82 originates from both NIP and decomposition products, but at the end of the isothermal evaporation experiment the decomposition products are the main molecular precursors of this ion.

The formation of bicyclic IL is not confirmed, because only one peak was registered in the GCMS experiment with a polar column and there were no traces of cyclization in NMR spectra.

To summarize, the vapor over BMImBF_4_ consists of NIPs and decomposition products according to routes **a** and **b**. Most of the previous investigations [17,18,19] used TGA and IR-spectroscopy to control the condensed phase of BMImBF_4_ and postulated the absence of any significant decomposition. No traces of these products was found from TGA and IR data on the condensed phase in our work. This discrepancy can be explained as follows. Analysis of the reported in literature [57,58,59,60,61] vapor pressures of potential dissociation products of IL under study (Figure 12) shows that they (with the exception of ylidene) are several orders of magnitude higher than those of prototypical IL BMImNTf_2_ [8]. Therefore, at experimental temperatures, these lightweight products cannot accumulate inside the effusion cell and they rapidly evaporate. That is why the IR-spectrum of the residue in an effusion cell is almost identical to that of the initial IL, whereas in the distillate collected from the cold parts of vacuum chamber the peaks attributed to imidazole-2-ylidene increased. The assessed vapor pressure of imidazole-2-ylidene is about one order of magnitude higher than that of IL. It leads to an amount of these vapor species becomes the higher the nearer are the evaporation conditions to equilibrium (closed system). 

The differences in vapor composition under Knudsen and Langmuir conditions, as demonstrated in [12], can be explained by the kinetically hindered decomposition of IL. In a closed system (Knudsen cell) the evaporation flux is in equilibrium with the reverse flux from the cell walls. The highly volatile decomposition products accumulate inside the effusion cell and their pressure become considerable. The reverse flux is absent under Langmuir conditions, leading to the decrease in the pressure of the decomposition products, which is limited by the hindered decomposition speed.

## 6. Conclusions

The evaporation of BMImBF_4_ IL is characterized by a complex vapor composition which leads to the appearance of atypical ions in its EI mass spectrum at much lower temperatures (424–514 K) than decomposition temperatures obtained by the TGA (685 K) method. Combined analysis of the KEMS, GCMS, NMR, and IR-spectroscopy data together with a thermal analysis and quantum chemical modelling reveal three competing routes of BMImBF_4_ evaporation: (1) congruent in the form of NIPs; (2) with decomposition in the form of imidazole-2-ylidene and HF; and (3) with decomposition in the form of 1-methylimidazole, 1-butene, HF, and BF_3_. Two other possible routes of decomposition of BMImBF_4_ in the form of bicyclic IL and H_2_ as well as 1-butylimidazole, CH_3_F, and BF_3_ are found to be negligible. Quantitative analysis of the vapor composition and vaporization thermodynamics will be given in future papers.

The vapor composition of BMImBF_4_ strongly depends on the evaporation conditions. Under equilibrium conditions (Knudsen cell), decomposition products prevail in vapor, while under Langmuir conditions (open surface), evaporation in the form of NIP is preferred. Vapor composition is temperature-dependent as well: the amount of [BMIm^+^][BF_4_^−^] NIPs relative to that of the decomposition products decreases by about a factor of three in the temperature range from 450 K to 510 K. The main reason for this specific evaporation of BMImBF_4_ is a high reactivity of the C1 atom in the imidazole ring, together with the high electronegativity of the anion. Similar peculiarities were observed for BMImPF_6_ evaporation and can be expected for all alkylimidazolium ILs with anions like BF_4_^−^, PF_6_^−^, AsF_6_^−^, SCN^−^, etc.

## Figures and Tables

**Table 1:** Ion appearance energies *AE* and coefficients *a* of the ln(*I∙T*) = −1000∙*a*/*T* + *b* dependency at *T* = 488 K.

*m*/*z*	*AE*, eV	*a*	Assigned Ion
49	16.9 ± 0.5	12.970 ± 0.274	BF_2_^+^
82	13.0 ± 0.5	17.486 ± 0.364	MIm^+^
96	11.3 ± 0.5	17.365 ± 0.442	MMIm^+^
137	11.0 ± 0.5	17.327 ± 0.455	C_8_H_13_N_2_^+^
139	12.4 ± 0.5	14.249 ± 0.390	BMIm^+^
158	11.7 ± 0.5	13.445 ± 0.363	BMImF^+^
187	13.8 ± 0.5	17.968 ± 0.510	BMImBF_2_^+^

A standard uncertainty is given with the “±” sign.

**Table 2:** Mass spectra of BMImBF_4_ recorded at different evaporation conditions.

	*T*, K	*m*/*z*					
		82	96	137	139	158	187
Effusion cell	487	250	455	95	100	18	68
Open cell	470	157	284	63	100	**	-
Open surface	471	52	52	21	100	**	-
Open surface *	501	44	23	15	100	13	-

*—reproduced as well as possible from Figure 3 in [14]; **—not measured.

**Table 3:** The relative intensities of four main peaks in mass spectra of BMImBF_4_ and imidazole-2-ylidene.

	Compound	*T*, K	*m*/*z*			
			82	96	137	187
GCMS (nonpolar column)	BMImBF_4_	523	34	100	18	19
KEMS (effusion cell)	BMImBF_4_	487	55	100	21	15
DIMS [10]	Imidazole-2-ylidene	323	28	100	27	64

**Table 4:** List of the main peaks in mass spectra [56] according to different decomposition processes (for a, b, c, and d designations see Figure 1).

Process	The Main Peaks with *m*/*z*
BMImBF_4(s)_ = BMImBF_4(g)_	82, 139, 158
a	82, 96, 137, 187
b	20, 28, 41, 42, 49, 54, 56, 81, 82
c	33, 34, 49, 81, 82, 97
d	137

## Data Availability

The data presented in this study is available in article.

## Supplementary Materials

The following are available online at https://www.mdpi.com/article/10.3390/e23111478/s1, Figure S1: Structures of BMImBF_4_ conformers, Figure S2. Experimental and theoretically predicted NMR spectra.
