# Supplementary material for: The Composition of Saturated Vapor over 1-Butyl-3-methylimidazolium Tetrafluoroborate Ionic Liquid: A Multi-Technique Study of the Vaporization Process
Source: Entropy (Basel). 2021 Nov 8;23(11):1478. doi: 10.3390/e23111478 (PMC8625100; doi:10.3390/e23111478)
Supplement: Supplementary file 1 [file entropy-23-01478-s001.zip › entropy-1430968-supplementary.pdf]

Supplementary materials to

To the question of the composition of saturated vapor over 1-butyl-3-methylimidazolium tetrafluoroborate ionic liquid: a multi-technique study of the vaporization process

A.M. Dunaev[1], V.B. Motalov, Ya.A. Radchenko, L.S. Kudin

Research Institute of Thermodynamics and Kinetics, Ivanovo State University of Chemistry and Technology, Ivanovo 153000, Russia

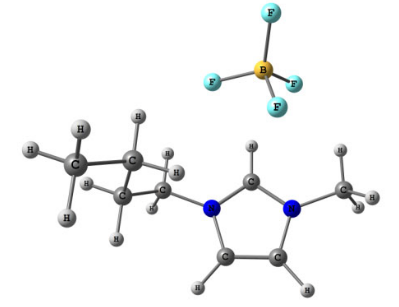	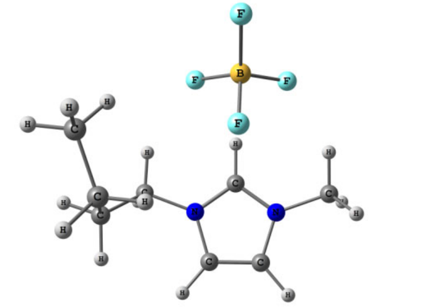	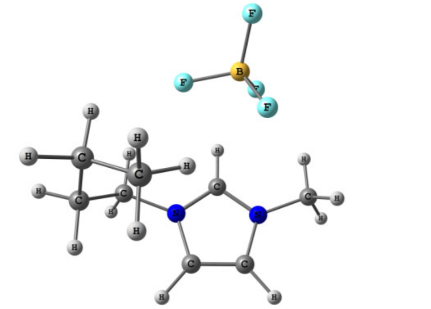
**1a** (0.001527)	**2a** (0.00284)	**3a** (0.004878)
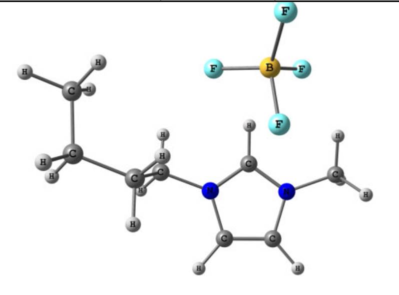	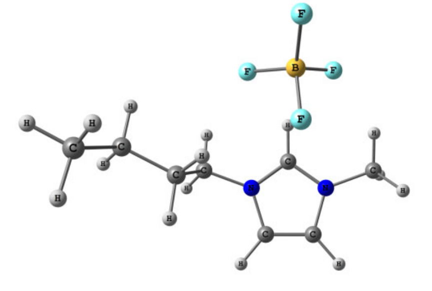	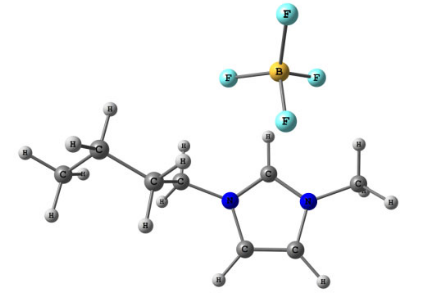
**4a** (0.000938)	**5a (0)**	**6a** (0.001768)
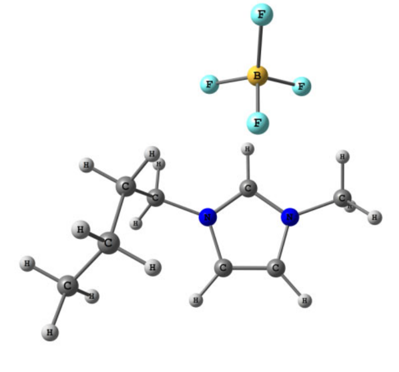	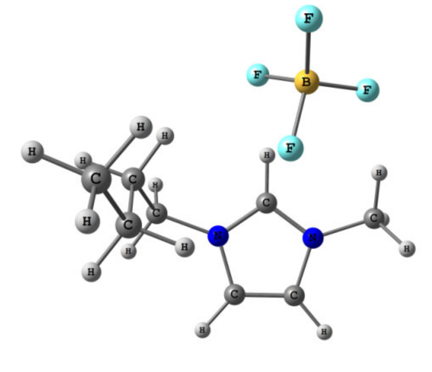	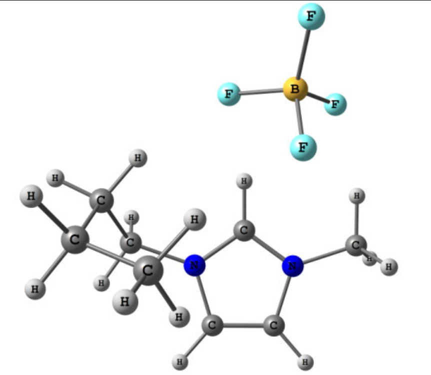
**7a** (0.003671)	**8a** (0.001288)	**9a** (0.00487)
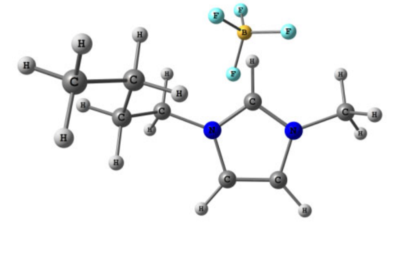	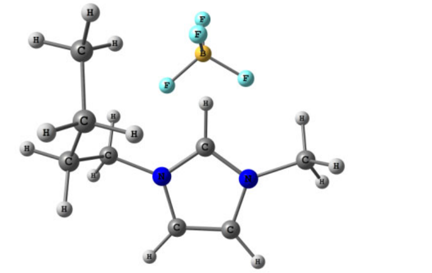	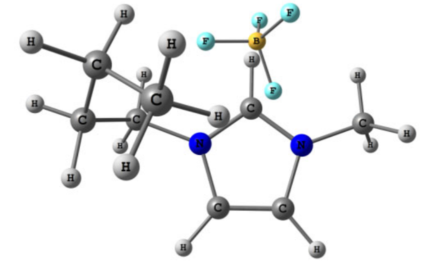
**1b** (0.000789)	**2b** (0.002645)	**3b** (0.005381)

[1] - corresponding author: amdunaev@ro.ru

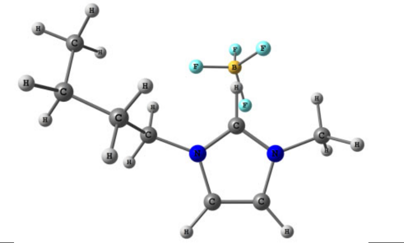	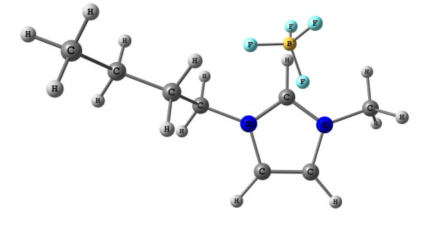	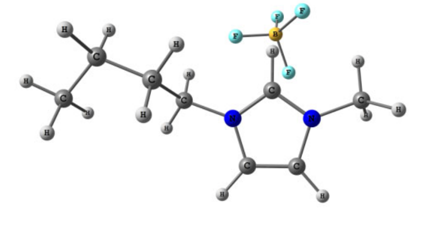
**4b (**0.00089**)**	**5b (**0.000503**)**	**6b (**0.002101**)**
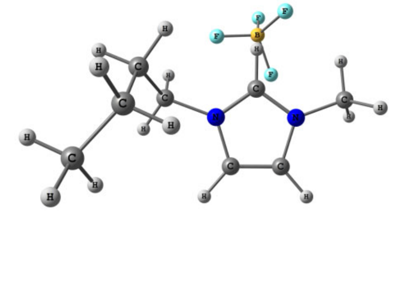	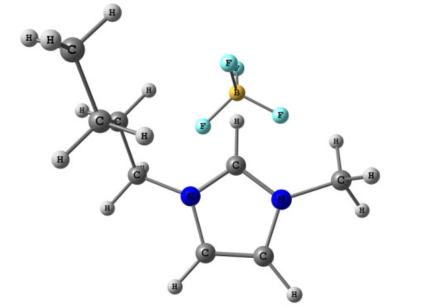	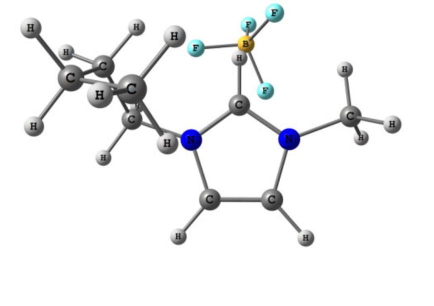
**7b (**0.002374**)**	**8b (**0.002397**)**	**9b (**0.004857**)**

Figure S1. Structures of $BMImBF_4$ conformers. Relative energies (hartree) are given in parentheses

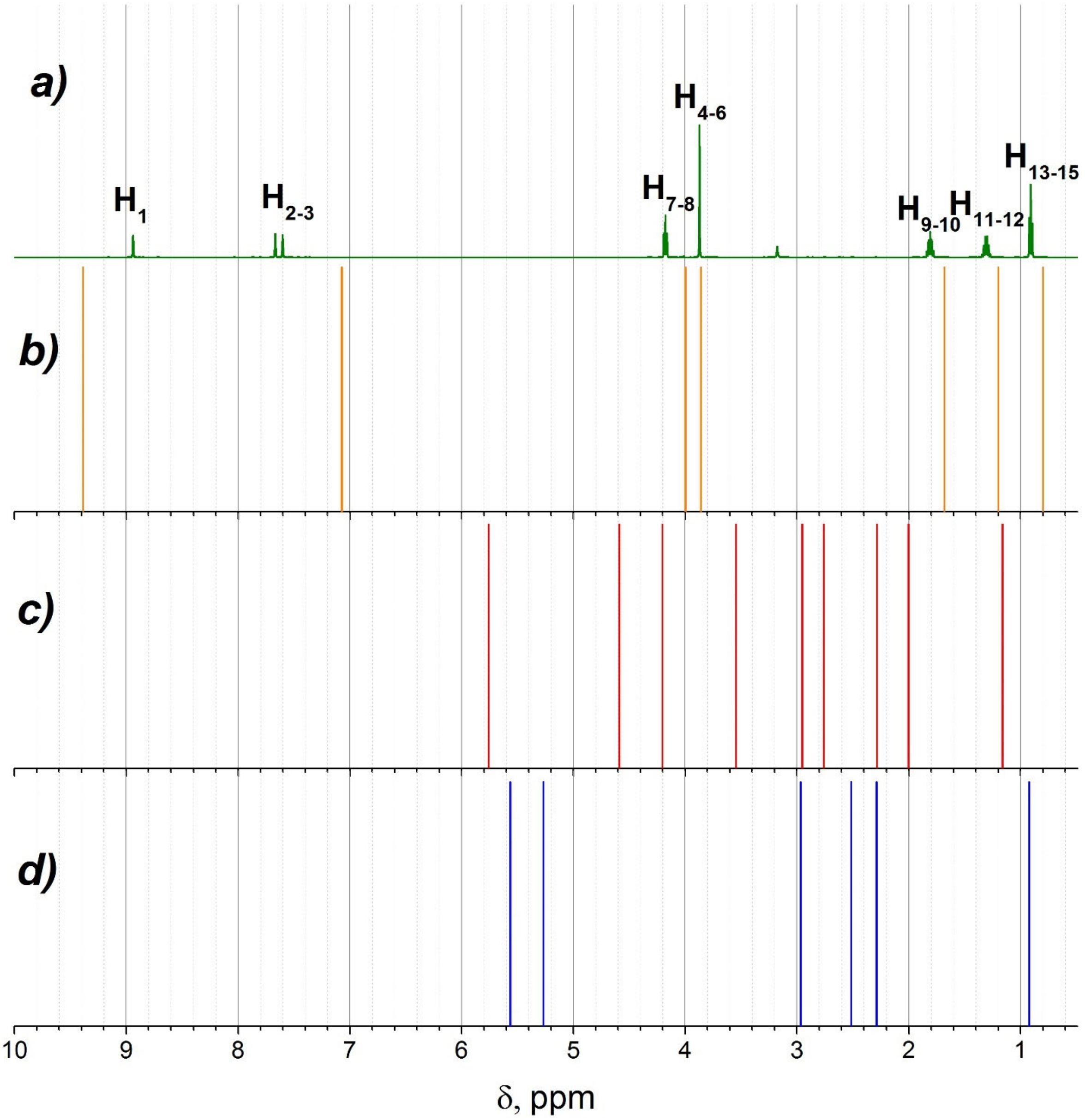

Fig. S2. Experimental *(a,* line) and theoretically predicted (*b*-*d,* bar) NMR spectra: *a*, *b* – $BMImBF_4$; *c* – imidazole-2-ylidene; *d* – bicyclic IL (see text)
